# Accountable or not accountable: A profile comparison of alleged offenders referred to the Free State Psychiatric Complex Forensic Observation Ward in Bloemfontein from 2009 to 2012

**DOI:** 10.4102/sajpsychiatry.v23i0.1054

**Published:** 2017-05-31

**Authors:** Edwin D. du Plessis, Henri J. du Plessis, Henco C. Nel, Inge Oosthuizen, Suzahn van der Merwe, Stefan Zwiegers, Gina Joubert

**Affiliations:** 1 Department of Psychiatry (G66), Faculty of Health Sciences, University of the Free State, South Africa; 2 School of Medicine, Faculty of Health Sciences, University of the Free State, South Africa; 3 Department of Biostatistics, Faculty of Health Sciences, University of the Free State, South Africa

## Abstract

**Background:**

The crime rate in South Africa is extraordinarily high. The problem of crime is further complicated when a person, who suffers from a mental illness, becomes involved in a crime. Furthermore, the forensic evaluation of a person suspected of having a mental illness involved in alleged criminal behaviour can be challenging. However, a dearth of information exists in South African literature regarding the link between crime and mental illness.

**Aim:**

To determine the percentage of alleged offenders, referred to the Free State Psychiatric Complex (FSPC) for observation, found accountable and not accountable, and to compare the biographical, diagnosis and offence profiles of these two groups. The analysis of differences can contribute to a better understanding of the complex process of forensic assessments.

**Setting:**

Forensic Observation Ward, FSPC, Bloemfontein.

**Methods:**

In this comparative, retrospective study, all 505 trial-awaiting alleged offenders (observati) referred from 2009 to 2012 for a 30-day observation period, according to Sections 77 and/or 78 of the Criminal Procedures Act, were included. Results were summarised as frequencies and percentages, and means or percentiles. Significant differences between the groups were determined by sample *t*-tests or chi-squared tests.

**Results:**

Observati found not accountable were in the majority (64.5%). Significant differences were found regarding marital and employment status, substance abuse, type of offence and diagnoses between the two groups. Almost all of the observati found to be not accountable were diagnosed with mental illness at the time of the assessment, whereas most observati found to be accountable for their actions at the time of the alleged offence were not found to be mentally ill. Observati found not accountable were significantly more likely to be diagnosed with schizophrenia, intellectual disability and substance-induced psychotic disorder, and committed mostly assault, murder and vandalism. Observati found accountable committed mostly rape, murder and theft.

**Conclusion:**

The majority of observati were found not accountable, with significant differences found between the two groups regarding demographic characteristics, type of diagnosis and offences committed. The identified differences can be used to assist in establishing criteria for the appropriate referral of alleged offenders by courts. Unnecessary referrals have a serious financial impact on the Department of Health. Furthermore, the high incidence of substance abuse among persons referred to the FSPC highlights the need for more substance rehabilitation centres in the Free State Province.

## Introduction

A high crime rate has a detrimental effect on the social well-being of a society. The problem is further complicated when a person suffering from a mental illness becomes involved in crime. Mental illness may deprive persons of the capacity to appreciate the unlawfulness of their conduct.^1,2^ It may also deprive them of the capacity to control their conduct.^2^ Sirotich^3^ published a research literature review in 2008 on the relationship between crime and violence, and mental disorders. The review looked at different variables of violence and crime among persons with mental illness, including gender, age, race, socio-economic status, history of violence or criminality, and substance abuse. National and international studies on forensic psychiatric patients showed that they were mostly unemployed, single men in their early thirties, with a history of substance abuse. A high percentage of patients were diagnosed with schizophrenia.^4,5,6^

South African courts can request an evaluation of the mental state of a person charged with committing a crime to determine whether the individual has any current mental problem that would interfere with his or her ability to participate in court proceedings.^7^ This enquiry is commonly interpreted as ‘fitness to stand trial’ or ‘competency to stand trial’ and is usually conducted by psychiatrists and clinical psychologists.^7,8,9^ If an accused is found not mentally ill, the court proceedings take their normal course. However, if the accused is evaluated as mentally ill, or if there is doubt concerning the accused’s mental state at the time of the offence (accountability or criminal responsibility), the court will order that the accused be observed in a designated psychiatric facility for a period not exceeding 30 days.^10,11^ Chapter 13 of the *Criminal Procedures Act* 51 of 1977^10,11^ contains provisions for this referral, and consists of three sections, Section 77 (trialability), Section 78 (accountability) and Section 79 (psychiatric assessment procedures). On completion of a psychiatric evaluation by psychiatrists or multidisciplinary mental health teams, an accused can either be found accountable for his or her actions, or not accountable as a result of a mental illness or defect. An accused found not accountable is declared a state patient by the court, and is admitted to a mental health institution, such as the Free State Psychiatric Complex (FSPC).

### Aim

The researchers sought to determine the percentage of alleged offenders referred to the FSPC for observation from January 2009 to December 2012, found accountable and not accountable, and to compare the biographical, diagnosis and offence profiles of these two groups.

## Research methods and design

### Definition of terms

Accountability – according to Section 78 (accountability) of the *Criminal Procedures Act* 51 of 1977 and Amendment 1998 – ‘the ability of the accused to appreciate the wrongfulness of his or her actions and whether he or she can act in accordance with an appreciation of the wrongfulness of his or her actions’.^10,11^

Observati – refers to the alleged offenders who are sent to a designated psychiatric facility for a 30-day observation period, according to Sections 77 and/or 78 of the *Criminal Procedures Act* 51 of 1977, to determine their mental state, accountability and triability.^12^

### Study design

This was a comparative, retrospective study.

### Setting

Forensic Observation Ward, FSPC, Bloemfontein.

### Study population and sampling strategy

All trial-awaiting alleged offenders (henceforth referred to as observati), sent to the Forensic Observation Ward of the FSPC from January 2009 to December 2012 (± 100 patients admitted per year) for a 30-day observation period in terms of Sections 77 and/or 78 of the Criminal Procedures Act, were included in this study.

## Ethical considerations

The study was approved by the Ethics Committee, Faculty of Health Sciences, University of the Free State. Permission to collect and analyse the data was obtained from the Chief Executive Officer of the Free State Psychiatric Complex (FSPC). None of the files was removed from the premises of the FSPC. The names of the observati were not recorded on the data forms, and all results and information were handled confidentially.

## Inclusion and exclusion criteria

No observations were excluded.

### Intervention

None.

### Data collection

Data were captured on a self-compiled data form, and included relevant information from clinical records and psychiatric reports. The forms were completed by the researchers in pairs to lower the risks of transcription errors.

### Pilot study

A pilot study was conducted by randomly selecting 10 files from the FSPC Forensic Observation Ward. These files were evaluated for completeness and utility to identify and eliminate possible problems. All the files used in the pilot study were included in the main study.

### Data analysis

Data were analysed by the Department of Biostatistics, Faculty of Health Sciences, University of the Free State (UFS). The results were summarised as frequencies and percentages (categorical variables), and means or percentiles (numerical variables). Independent sample *t*-tests, chi-squared tests or Fisher’s exact tests with 95% confidence intervals (CIs) were used to determine significant differences between the two groups (observati found accountable versus observati found not accountable).

## Results

After the 30-day observation period at the FSPC Forensic Observation Ward, the multidisciplinary team found almost two-thirds (64.6%) of the 505 observati unaccountable (Table 1).

**TABLE 1:** Outcomes of the observati after the 30-day observation period.

Outcome	Total study population (*n* = 505)	

	*N*	%
Accountable	169	33.5
Not accountable	326	64.6
Unknown	10	2.0

The majority of the observati in both groups (> 94%) were men (Table 2). The highest percentage of observati in both groups was between the ages of 20 and 29 years at the time of the offences. More than 75% of the observati in both groups were Sotho-speaking.

**TABLE 2:** Socio-demographic data of observati at the time of the offence.

Variable	Accountable		Not accountable		*p*
	
	*n*	%	*n*	%	
**Age groups (years)**	**(*n* = 169)**		**(*n* = 323)**		**0.64**
9–19	16	9.5	15	4.6	
20–29	58	34.3	139	43.0	
30–39	48	28.4	90	27.9	
40–49	24	14.2	56	17.3	
50–59	14	8.3	15	4.6	
60–69	5	3.0	6	1.9	
> 70	4	2.4	2	0.6	
**Home language**	**(*n* = 163)**		**(*n* = 309)**		**0.38**
Afrikaans	20	12.3	27	8.7	
English	4	2.5	4	1.3	
Sotho	123	75.5	247	79.9	
Zulu	1	0.6	5	1.6	
Tswana	9	5.5	12	3.9	
Xhosa	5	3.1	14	4.5	
Bangli	1	0.6	0	0	
**Gender**	**(*n* = 169)**		**(*n* = 326)**		**1.00**
Male	160	94.7	308	94.5	
Female	9	5.3	18	5.5	
**Marital status**	**(*n* = 157)**		**(*n* = 306)**		**0.02**
Married	16	10.2	18	5.9	
Single	121	77.1	272	88.9	
Divorced	5	3.2	5	1.6	
Living together	11	7.0	8	2.6	
Other	4	2.6	3	1.0	
**Employment status**	**(*n* = 164)**		**(*n* = 314)**		**< 0.01**
Employed	49	29.9	35	11.2	
Unemployed	79	48.2	175	55.7	
Disability grant	28	17.1	90	28.7	
Scholar	3	1.8	6	1.9	
Pensioner	5	3.1	7	2.2	
Vendor	0	0	1	0.3	

Statistically significant differences in the marital and employment statuses were found between the two groups. More than three-quarters (77.1%) of observati found accountable were single compared with 88.9% of observati found not accountable.

Only 11.2% of non-accountable observati were employed compared with 29.9% of observati found accountable. At least 70.0% of observati in both groups were unemployed (which include observati receiving disability grants, scholars and pensioners).

According to Table 3, more than 66% of observati in both groups had a known history of substance abuse. There was no statistically significant difference in history of substance abuse between the two groups.

**TABLE 3:** History of substance abuse and the substance use of the observati at the time of the offence.

Variable	Accountable		Not accountable		*p*
	
	*n*	%	*n*	%	
**History of substance abuse**	**(*n* = 148)**		**(*n* = 303)**		**0.67**
Yes	98	66.2	207	68.3	
No	50	33.8	96	31.7	
**Substance use at the time of the offence**	**(*n* = 94)**		**(*n* = 140)**		
None	29	30.9	47	33.6	0.66
Alcohol	42	44.7	47	33.6	0.09
Cannabis	12	12.8	29	20.7	0.12
Alcohol + cannabis	7	7.5	14	10.0	0.50
Cannabis + glue	1	1.1	2	1.4	1.00†
Glue	2	2.1	0	0	0.16†
Alcohol + cocaine	1	1.1	0	0	1.00†
Alcohol + cannabis + glue	0	0	1	0.7	0.40†

† , Fisher’s exact tests were used because of small numbers.

Of those with available data (53.3%), 65 of the 94 (69.1%) observati found accountable and 93 of the 140 (66.4%) observati found not accountable were under the influence of some kind of substance at the time the offences were committed. In both groups, alcohol was the substance most used at the time of the offence (accountable observati = 44.7% and not accountable observati = 33.6%). A higher percentage (20.7%) of observati found not accountable used cannabis compared with 12.8% of observati found accountable. Offences committed by the observati against persons and property are summarised in Tables 4 and 5.

**TABLE 4:** Offences committed by observati against persons.

Offence committed against persons	Accountable (*n* = 169)		Not accountable (*n* = 326)		*p*
	
	*n*	%	*n*	%	
Murder	37	21.9	32	9.8	< 0.01
Attempted murder	15	8.9	12	3.7	0.02
Assault	21	12.4	113	34.7	< 0.0001
Rape	64	37.9	69	21.2	< 0.0001
Attempted rape	5	3.0	21	6.4	0.10
Sexual offences other than rape	5	3.0	8	2.5	0.77†
Sodomy	0	0	1	0.3	1.00†
Contravention of protection order	3	1.8	12	3.7	0.24
Fraud	6	3.6	1	0.3	0.01†
Dealing with dagga	1	0.6	0	0	0.34†
Child abuse	1	0.6	0	0	0.34†
Domestic violence	1	0.6	2	0.6	1.00†
Defeating the end of justice	1	0.6	0	0	0.34†
Conspiracy to commit murder	1	0.6	0	0	0.34†
Intimidation	1	0.6	1	0.3	1.00†
Resisting arrest	1	0.6	0	0	0.34†
Culpable homicide	2	1.2	1	0.3	0.27†
Concealment of birth	0	0	1	0.3	1.00†

† , Fisher’s exact tests were used because of small numbers.

**TABLE 5:** Offences committed by observati against properties.

Offence against property	Accountable (*n* = 169)		Not accountable (*n* = 326)		*p*
	
	*n*	%	*n*	%	
Vandalism	7	4.1	34	10.4	0.02
Theft	20	11.8	31	9.5	0.42
Burglary	14	8.3	32	9.8	0.58
Robbery	14	8.3	5	1.5	< 0.01
Arson	1	0.6	8	2.5	0.18†
Possession of suspected stolen property	1	0.6	0	0	0.34†
Fraud, forgery and uttering	1	0.6	0	0	0.34†
Bestiality	0	0	1	0.3	1.00†
Trespassing	0	0	4	1.2	0.31†

† , Fisher’s exact tests were used because of small numbers.

The most common offences committed against persons by observati found accountable were rape (37.9%) and murder (21.9%). Assault (34.7%) and rape (21.2%) were the most common offences committed against persons by observati found not accountable. In observati found accountable, 43.8% committed sexual offences compared with the 30.4% observati found not accountable.

Statistically significant differences were seen in the main offences committed against persons by the two groups. Observati committing sexual offences, including rape, were mostly found accountable.

Theft (11.8%) was the most common offence committed against properties by observati found accountable. For observati found not accountable, vandalism (10.4%) was the top offence. Statistically significant differences were noted for vandalism and robbery. The diagnoses of observati found accountable and not accountable are shown in Table 6.

**TABLE 6:** Diagnoses of the observation.

Diagnosis	Accountable (*n* = 146)†		Not accountable (*n* = 305)†		*p*
	
	*n*	%	*n*	%	
No mental illness	92	63.0	6	2.0	0.0001
Schizophrenia	15	10.3	193	63.3	0.0001
Mental retardation	5	3.4	51	16.7	0.0001
Bipolar mood disorder	6	4.1	11	3.6	0.79
Psychosis due to a general medical condition	3	2.1	16	5.2	0.11
Psychosis due to epilepsy	2	1.4	5	1.6	0.83
Substance-induced psychotic disorder	4	2.7	26	8.5	0.02
Delirium	0	0	2	0.7	1.00‡
Personality disorder	2	1.4	0	0	0.10‡
Dementia	6	4.1	8	2.6	0.40‡
Amnesia	2	1.4	0	0	0.10‡
Folic acid deficiency	0	0	1	0.3	1.00‡
Epilepsy	5	3.4	8	2.6	0.76‡
Axis 2 personality disorder	1	0.7	0	0	0.32‡
Post-traumatic stress disorder	2	1.4	1	0.3	0.25‡
Psychosis	3	2.1	15	4.9	0.15
Frontal lobe syndrome + epilepsy	0	0	1	0.3	1.00‡
Conduct disorder	0	0	1	0.3	1.00‡
Major depressive disorder	0	0	1	0.3	1.00‡
Psychotic disorder	0	0	1	0.3	1.00‡
Temporal lobe epilepsy	0	0	1	0.3	1.00‡
Pseudologia fantastica factitious disorder	0	0	1	0.3	1.00‡

† , Diagnosis was not traceable in 23 and 21 of the observation per group, respectively.

‡ , Fisher’s exact tests were used because of small numbers.

Nearly two-thirds (63.0%) of the observati found accountable had no mental illness, compared with only 2.0% of observati found not accountable (95% CI for difference 52.7% – 68.5%). Statistically significant differences for schizophrenia, mental retardation and substance-induced psychotic disorder were found between the two groups. The majority (63.3%) of observati found not accountable were diagnosed with schizophrenia compared with 10.3% of observati found accountable. Mental retardation was diagnosed in 16.7% of observati found not accountable compared with only 3.4% of observati found accountable.

At the time of the offence, 38.5% of observati found accountable and 28.5% of observati found not accountable were under the influence of a substance. In our study population, 31 observati were diagnosed with substance-induced psychotic disorder, of which 4 (2.7%) were found accountable and 26 (8.5%) were found not accountable.

## Discussion

Almost two-thirds (64.5%) of the observati at FSPC were found unaccountable. This is lower than the 80% found by Strydom et al.,^6^ but in line with findings by Wang et al.^5^ (64.1%). The percentage of the observati found accountable (33.5%) is in contrast to the data from a similar study performed between 1995 and 2001 at the FSPC, where nearly half of observati (48.6%) were found accountable.^13^

The majority of the observati in both groups were men. Similar percentages were reported by Douglas et al.^4^ (91.0%), Wang et al.^5^ (87.7%) and Strydom et al.^6^ (95.8%). In his literature review, Sirotich^3^ noted that some studies considered male gender an important predictor of violent or criminal behaviour among persons with mental disorders. On the contrary, studies in psychiatric patients found that men were no more prone to violence than women were. Observati in both groups were in their early twenties at the time of the offence. Persons in their late teens and early twenties suffering from mental illness are at the highest risk for violent or criminal behaviour.^3^

Race was not always recorded in the files, and the researchers used language as a variable to get insight into the race distribution of the observati. More than three-quarters of the observati in both groups were Sotho-speaking. Strydom et al.^6^ also found that 75% of their study population spoke Sotho. Studies of mentally ill offenders have shown a link between race and violence, but the effect of race seems to be lessened by other factors.^3^

Significantly more observati found not accountable were single. The majority of participants studied by Douglas et al.^4^ (67.0%), Wang et al.^5^ (53.5%) and Strydom et al.^6^ (83.8%) were also single.

Most (70.0%) of the observati were unemployed. The percentage of employed observati in the non-accountable group was much lower than the percentage in the accountable group. Strydom et al.^6^ reported an unemployment rate of 81.5%; both results are much higher than the unemployment rate in South Africa during 2009 to 2012, which varied between 23.4% and 25.7%.^14^

About two-thirds of both groups reported a history of substance abuse; however, there was no significant difference between the two groups. The results are similar to findings of Verster and Van Rensburg^15^ who reported that, of the offenders charged with murder, 77% had a history of alcohol abuse and 36% of cannabis abuse.

In more than half of the cases, substance abuse at the time of the offence was not reported. This can be problematic when the accountability versus not accountability comes into play, seeing that the substances used at the time of the offence may play a major role in determining the observati’s mental state at that time, and thus the accountability of the observati. Alcohol was the primary substance used at the time of the offence, followed by cannabis. Mosotho et al.^16^ also reported that alcohol was the most commonly abused substance among the Sotho-speaking population in Mangaung, Bloemfontein, followed by nicotine and cannabis.

Observati found accountable mostly committed rape and murder, whereas assault and rape were the most common offences committed by observati found not accountable. This is in line with findings by Strydom et al.^6^ where most of the offences (80.0%) committed by observati referred to the FSPC during 2004 to 2008 were against persons, and included rape, assault and murder.

Observati committing sexual offences were typically found accountable. This was also true to a lesser extent for murder and attempted murder. Joubert and Van Staden^17^ reported that 62.6% of observati accused of murder and sent to Weskoppies Hospital for observation were found criminally responsible.

Almost all of the observati found not accountable had a mental illness diagnosis. In contrast, most observati found accountable did not have any mental illness. Similar results were reported by Gagiano et al.^18^ (52.0%), Slabber et al.^19^ (42.0%) and Verster and Van Rensburg^15^ (57.9%).

Observati found not accountable were significantly more likely to be diagnosed with schizophrenia, mental retardation and substance-induced psychotic disorder. Similar findings were reported by Slabber et al.^19^ (21.0%), Verster and Van Rensburg^15^ (20.0%) and Pretorius et al.^20^ (23.3%).

## Conclusion

In the time span from 2009 to 2012, 505 observati were sent by the court for observation to the FSPC Forensic Observation Ward. After the 30-day observation period, 64.5% were found not accountable, whereas 35.5% were found accountable.

Socio-demographically, the two groups were similar with regard to age, home language and gender distribution. Observati found not accountable were more likely to be single and unemployed than observati found accountable. In both groups, the majority of observati had a history of substance abuse. This confirms that, regardless of the presence of mental illness, substance abuse is still a major factor in violent and criminal behaviour in South Africa.

Observati found not accountable were significantly more likely to be diagnosed with schizophrenia, intellectual disability and substance-induced psychotic disorder, and committed mostly assault, murder and vandalism. Observati found accountable committed mostly rape, murder and theft. Mental conditions such as schizophrenia, mental disability and psychosis may deprive, even though not necessarily, persons of the capacity to appreciate the unlawfulness of their conduct. It may also deprive them of the capacity to control their conduct. It is not surprising that all of the persons found not accountable suffered from the symptoms of one or more of these conditions at the time of the offence. The high incidence of serious offences such as assault, murder and rape among this sample supports the notion that the incidence of serious offences in South Africa is on the rise.

There were significant differences in the two groups regarding marital status, employment status, offences against persons, offences against property and diagnosis. However, more research is needed to make definitive conclusions.

Although this study provided noteworthy findings, the results should be interpreted with care, especially as far as their generalisation is concerned. Only observati at the FSPC, and therefore from its catchment area, were included in the study. Furthermore, not all the possibly mentally ill observati were referred by the courts for observation. Some of the files were incomplete with important data missing. Nevertheless, this study contributes important data regarding demographics, psychiatric diagnoses and offence profiles in the field of forensic psychiatry.
